# Auscultation of Velcro Crackles is Associated With Usual Interstitial Pneumonia

**DOI:** 10.1097/MD.0000000000002573

**Published:** 2016-02-08

**Authors:** Jacobo Sellarés, Fernanda Hernández-González, Carmen Mª Lucena, Marina Paradela, Pilar Brito-Zerón, Sergio Prieto-González, Mariana Benegas, Sandra Cuerpo, Gerard Espinosa, José Ramírez, Marcelo Sánchez, Antoni Xaubet

**Affiliations:** From the Servei de Pneumologia, Hospital Clínic, IDIBAPS, Universitat de Barcelona, (JS, FH-G, CMªL, SC, AX); Centro de Investigación Biomedica En Red-Enfermedades Respiratorias (CibeRes, CB06/06/0028) (JS, CMªL); Servei de Cirurgia Toràcica, Hospital Clínic, IDIBAPS, Universitat de Barcelona (MP); Servei de Malalties Autoimmunes (PB-Z, SP-G, GE); Servicio de Radiodiagnóstico (MB, MS); and Servicio de Anatomía Patológica, Hospital Clínic, Barcelona, Spain (JR).

## Abstract

Auscultation of Velcro crackles has been proposed as a key finding in physical lung examination in patients with interstitial lung diseases (ILDs), especially in idiopathic pulmonary fibrosis (IPF). However, no studies have been carried out to assess the association of Velcro crackles with other clinical variables.

We evaluated a cohort of 132 patients, prospectively and consecutively included in our ILD diagnostic program at a tertiary referral center. All patients were auscultated during the physical examination. The patients were divided into 2 groups: “presence” or “nonpresence” of bilateral Velcro crackles.

Of all patients assessed, 83 (63%) presented Velcro crackles in the respiratory auscultation. Patients with Velcro crackles usually had more frequently cough and dyspnea at the moment of diagnosis. Forced vital capacity (*P* = 0.002) and lung diffusion capacity for carbon monoxide (*P* = 0.04) was lower in these patients. The ILD-GAP index was higher in the group with Velcro crackles (*P* = 0.01). All patients with usual interstitial pneumonia (UIP) in high-resolution computed tomography and all patients with final IPF diagnosis presented Velcro crackles. In multivariate analysis, the presence of Velcro crackles was independently associated with an UIP pattern.

In patients suspected of having ILD, the auscultation of Velcro crackles was associated with UIP, a possibility which must be taken into consideration in early ILD detection in primary care.

## INTRODUCTION

Diffuse interstitial lung diseases (ILDs) are a group of rare diseases characterized by inflammation and subsequent fibrosis of the lung parenchyma. The incidence of ILDs is estimated to be 26 to 32 cases per 100,000 inhabitants, and they are associated with high morbidity and mortality.^1^ The main problems in the diagnosis of ILDs are: (1) the insidious initiation of the disease with nonspecific symptoms; (2) the difficulty in establishing the origin of many ILDs; (3) the limited therapeutic options, especially when these diseases are advanced; (4) the necessity of advanced diagnostic tools (high resolution computed tomography, transbronchial biopsy, etc) and an experienced multidisciplinary team (usually only available in tertiary hospitals) to obtain an adequate diagnosis.

Early diagnosis and early referral to a specialized ILD program are essential in ILDs, especially in the case of idiopathic pulmonary fibrosis (IPF). Although no curative therapy is currently available, apart from lung transplantation, early diagnosis is the only option, as the objective of the current medication is to slow down the progression of the disease in most of the cases.^1^ Once the ILD is detected, any delay in referring the patient to a tertiary centre is associated with higher mortality, irrespective of disease severity.^2^

Different options have been proposed in order to increase the cases of early diagnosis in ILDs.^3,4^ The appearance of Velcro crackles in lung auscultation has been proposed as an early sign of IPF,^5^ in ILD associated with rheumatoid arthritis^6^ and asbestosis.^7^ However, no validated protocol has been proposed and these Velcro crackles may be not present in other ILD without pulmonary fibrosis.^5^ In addition, although the auscultation of Velcro crackles has been reported in different ILDs,^8^ there is no data indicating the association of Velcro crackles with clinical and radiological characteristics upon diagnosis.

Accordingly, the aim of our study was to assess the association of Velcro crackles with different clinical and radiological characteristics in a group of patients that were referred to a specialized ILD diagnosis program.

## MATERIAL AND METHODS

### Study Setting and Patient Population

A prospective observational study was carried out in the ILD unit from the Hospital Clínic of Barcelona. All patients suspected of having ILD that were referred to the diagnostic program of the ILD Unit during the period between September 2012 and July 2014 were consecutively and prospectively included in the study. The following diagnostic procedures were carried out during the study: complete clinical history and physical examination, conventional and high-resolution computed tomography (HRCT), blood analysis including different autoimmune markers, lung function test with blood arterial gas exchange analysis, 6-min walking test, and echocardiography. With this information, the patient was presented in our ILD multidisciplinary session and the potential need to carry out a biopsy to confirm diagnosis was discussed. All diagnoses were established following the standard criteria.^1,9–12^ ILD–GAP score classes were assigned in accordance with the authors’ original designations.^13^ ILD-GAP components are age, gender, forced vital capacity (FVC), and lung diffusion capacity for carbon monoxide (DLco) and type of ILD.

HRCT of the chest without intravenous contrast medium was performed during end inspiration with the patient in the supine position. The HRCT features of ILD included in the present study were “presence of bilateral reticular abnormalities,” “traction bronchiectasis,” and “honeycombing,” characteristics usually associated with fibrotic ILDs. In addition, usual interstitial pneumonia (UIP), possible UIP and inconsistent with UIP patterns were identified according to validated criteria.^10^ Other patterns from the group that was inconsistent with UIP were also identified: nonspecific interstitial pneumonia (NSIP), organizing pneumonia (OP), and lymphoid interstitial pneumonia (LIP).^1,14^ Images were read independently by 2-blinded radiologists (Dr. M Sánchez and M. Benegas). Discrepant readings were re-reviewed to determine consensus findings. The study protocol was approved by the Ethical Committee of the hospital.

### Velcro Crackles Auscultation

In the routine examination of the patients during the diagnostic protocol, respiratory auscultation was performed. Previously to auscultation, limited information was available from the patients (name, age, suspicion of an ILD). Clinical and radiological assessment was performed after physical examination. In addition, the auscultation was performed before the results of the different diagnostic tests and the multidisciplinary evaluation were known.

Bilateral auscultation of the lungs was performed from the upper regions to the basal areas. Velcro crackles were defined as bilateral crepitations, detected during slow, deep breaths, predominating during inspiration, and best heard over dependent lung regions, and sometimes associated with expiratory crackles, with a sound similar to the sound heard when gently separating the strip of velcro attached to the blood pressure cuff (or jogging shoes).^5,15^ The auscultation of Velcro crackles was performed and confirmed by 2 different physicians in all cases (J. Sellarés and F. Hernández-González). Patients were classified in 2 groups: “presence of bilateral Velcro crackles” or “nonpresence of bilateral Velcro crackles.”

### Statistical Analysis

Categorical variables were summarized with counts and percentages. For continuous variables, the mean and standard deviation (SD) were presented. Quantitative continuous variables were compared using Student's *t* test categorical variables were contrasted by the chi-square test.

Multivariate analyses were performed with logistic regression to assess the association of Velcro crackles with variables that were significantly associated in the univariate analysis. The variables included were: the presence of previous respiratory symptoms, cough, dyspnea, digital clubbing, FVC, DLco, UIP and possible UIP group and ILD-GAP index. UIP and possible UIP patients were combined in 1 group because UIP variable could not be included in the logistic regression, as 1 category (UIP without crackles) has no patients included and the multivariate analysis could not be performed in this case.^16^ A conditional stepwise forward model (*P*_in_ < 0.10, *P*_out_ < 0.05) was used to correct for co-linearity, and adjusted odds-ratios and 95% confidence intervals were computed for the outcome variables. The level of significance was set at 0.05. The statistical analyses were performed using IBM SPSS Statistics, Release 20, SPSS Inc., 2011.

## RESULTS

### Patient Characteristics

A total of 132 adult patients evaluated for suspected ILD were studied, of whom 83 (63%) presented Velcro crackles in the respiratory auscultation. No patients presented unilateral crackles when they were auscultated.

The baseline clinical characteristics of the study population are shown in Table 1, divided in 2 groups: patients with and without Velcro crackles. No significant differences were observed between both groups.

**TABLE 1:**
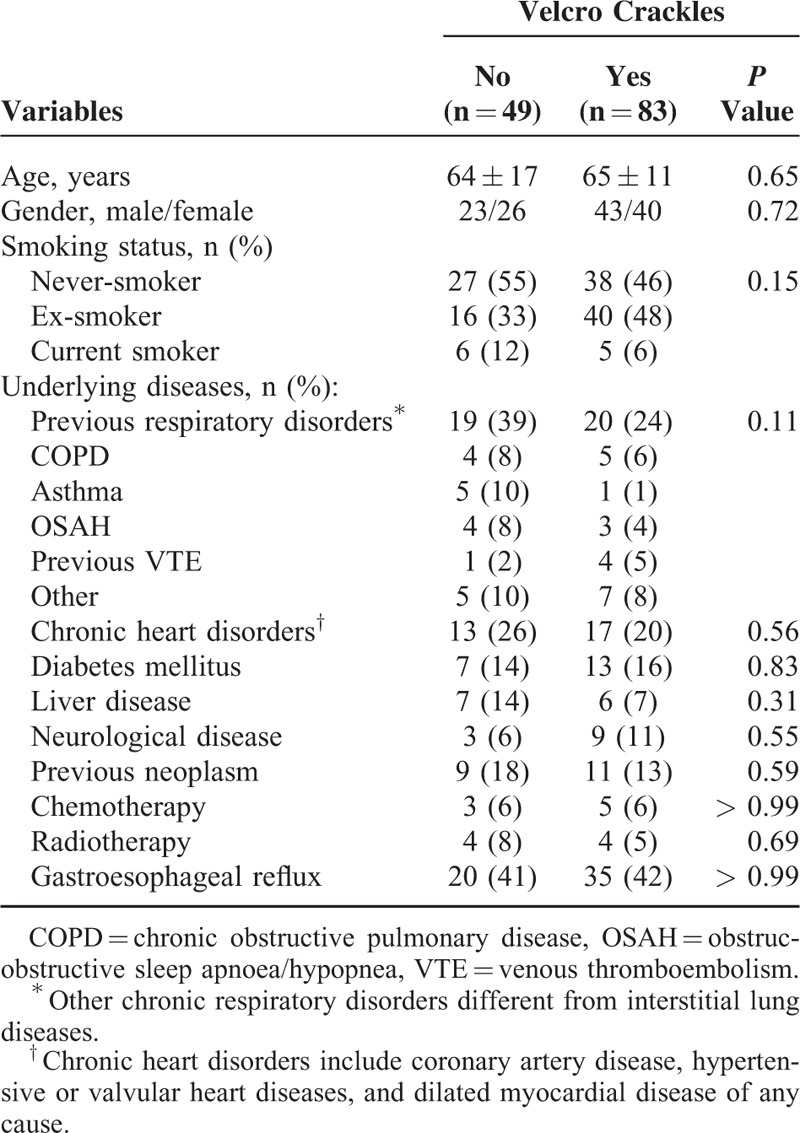
Baseline Characteristics of the Patients

Table 2 summarizes the signs and symptoms, functional and radiological data, and the ILD-GAP index of the patients. Patients with Velcro crackles usually had previous respiratory symptoms and more frequently cough and dyspnea at the moment of diagnosis. Pulmonary function was worst in these patients and they have more frequently radiological signs of honeycombing and reticular abnormalities. From all the radiological patterns assessed, UIP was associated with Velcro crackles auscultation. From the other different patterns included in the “inconsistent with UIP” group, no significant differences were observed, although NSIP had a tendency to present a higher frequency of Velcro crackles auscultation. The limited number of patients in OP and LIP make it difficult to interpret the association of these patterns with Velcro crackles. The ILD-GAP index was also higher in the group with Velcro crackles.

**TABLE 2:**
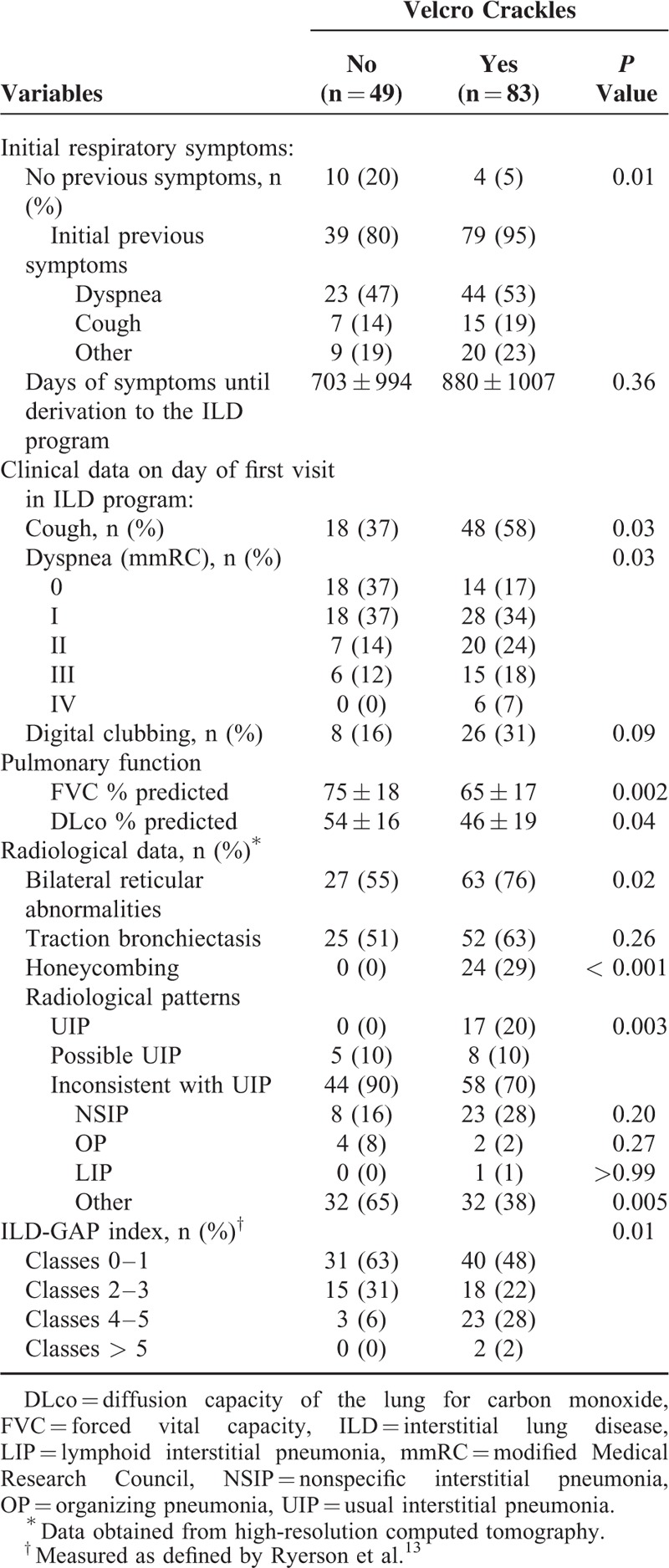
Signs and Symptoms, Functional and Radiological Data and the ILD-GAP Index

### ILD Multidisciplinary Diagnosis and Velcro Crackles Auscultation

Table 3 shows the different diagnoses according to which patients were classified after the multidisciplinary discussion. Patients with IPF and connective tissue disease-associated interstitial lung disease (CTD-ILD) had more frequently Velcro crackles at auscultation. In all the cases of IPF, the respiratory auscultation detected Velcro crackles. In the 13 patients with CTD-ILD and Velcro crackles, only 3 had honeycombing and 8 traction bronchiectasis.

**TABLE 3:**
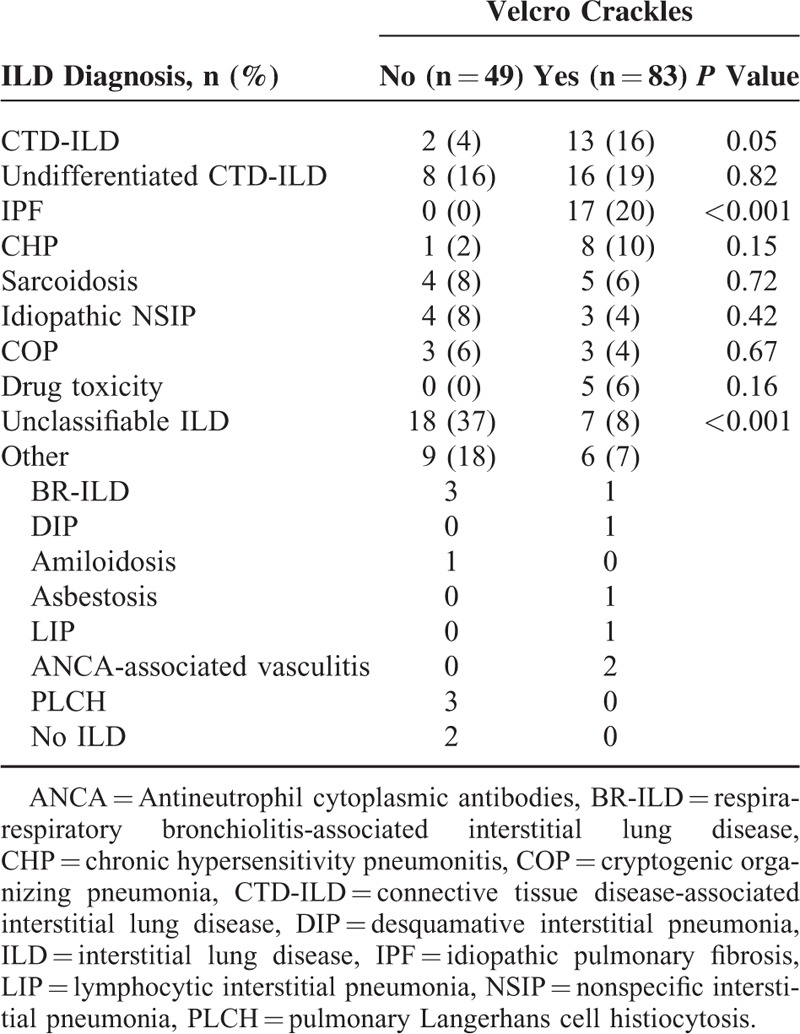
Diagnosis After Multidisciplinary Discussion

We also grouped patients with CTD together with undifferentiated CTD and reviewed the different radiological patterns observed. A total of 39 patients were analyzed and classified in “no Velcro Crackles auscultation” and “Velcro Crackles auscultation” groups, and the following results were obtained: NSIP (3 [30%] and 15 [52%]), UIP (0 [0%] and 3 [10%]), possible UIP (1 [10%] and 3[10%]), OP (3 [30%] and 0[0%]), LIP (0 [0%] and 1[3%]), and other (3 [30] and 7 [24]), *P* = 0.053. Thus, the auscultation of Velcro crackles in the CTD group seemed to be mainly related to UIP and NSIP patterns, although statistical analysis was not significant. Interestingly, OP pattern did not seem to be associated with Velcro crackles, although the limited sample made adequate interpretation not possible.

Of the 22 patients with unclassifiable ILD and Velcro crackles, only 1 had honeycombing and 2 traction bronchiectasis.

### Multivariate Analyses of Clinical Variables Associated With Velcro Crackles

In the multivariate regression analysis (Table 4), after adjusting for the potential confounders, Velcro crackles auscultation on the initial examination of an ILD program was independently associated with the presence of respiratory symptoms previous to initial visit and UIP/possible UIP pattern in HRCT scan.

**TABLE 4:**
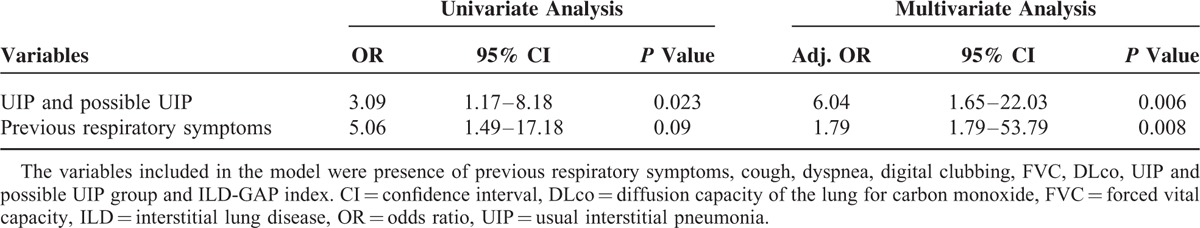
Multivariate Analysis of the Association of Velcro Crackles With Clinical and Radiological Variables

## DISCUSSION

The main findings of the study are that in patients with ILD suspicion, the auscultation of Velcro crackles is independently associated with the presence of UIP, which is described to be associated with a worst prognosis.^17^ When patients were separated by specific ILD, IPF and CTD-ILD more frequently have Velcro crackles at auscultation. In the case of IPF, all of the patients with a final diagnosis of IPF have Velcro crackles at auscultation.

Not many studies have assessed the significance of auscultation of Velcro crackles in ILDs. A “classic” 1978 study of Epler et al,^8^ observed fine or Velcro crackles in 60% of patients with ILD, a percentage similar to our ILD patients (63%). Other studies have shown that the auscultation of crackles is associated with preclinical ILD detection in rheumatoid arthritis (RA)^6^ and early detection of asbestosis.^7^ In a recent study,^18^ Velcro crackles were included as a fundamental part in different algorithms to detect ILDs in systemic sclerosis.

In our study we have observed that the auscultation of Velcro crackles in patients with ILD suspicion is independently associated with UIP, what is related to a worst prognosis.^17^ This is concordant with the fact that these patients also have more frequently respiratory symptoms previously to the diagnosis, as they were more severe. When assessing individually ILD-GAP components, only functional data and type of ILD were significantly associated with Velcro crackles (Tables 1–3). In conjunction, with these findings, we also have observed that the presence of honeycombing in HRCT is also associated with Velcro crackles. This association was also found in the previous study of Epler et al.^8^ Honeycombing is one of the key findings to define a UIP pattern in HCRT,^10^ what is associated with IPF^10^ and a worst outcome in ILD.^17^

Although Velcro crackles have been associated more clearly to UIP in our study, we have observed that crackles were also present in 58 patients from 102 with a pattern of inconsistent with UIP (Table 2). So, it is probable that in addition to the radiological UIP or non UIP-pattern, the determinant of the presence of Velcro crackles could be the “fibrotic” changes. This is consistent with previous publications^6–8,18^ and with the radiological findings of our study, as we found more typical radiological fibrotic changes in patients with crackles (radiological data, Table 2). On the other hand, it is possible that the NSIP pattern, with fibrotic changes, could also be linked to Velcro crackles, although in our study it was only possible to observe a tendency, particularly in CTD patients. The most likely mechanism for the generation of Velcro crackles is the sudden inspiratory opening of small airways held closed by surface forces during the previous expiration.^15^ At which point or level of fibrosis this phenomenon occurs is unknown. However, we could conclude from the results of our study that the presence of Velcro crackles is a common finding in a “pure fibrotic” manifestation as UIP, as all the patients with UIP presented crackles at auscultation, what suggests the relation of fibrosis-Velcro crackles. Further research is required before it will be possible to determine which subgroups of the NSIP pattern may be associated with Velcro crackles, although in our study this association appears to be more consistent with UIP than with NSIP.

Velcro crackles have been proposed as a sign for early diagnosis in IPF.^3–5,19^ However, no studies have validated the use of lungs auscultation in the early detection of IPF in primary care. In addition, the analysis of crackles sounds using a sound analyzer has been proposed as a promising tool in the ILDs assessment.^20^ However, we still did not have the available technology to apply these techniques in all clinical settings. In our study, all the patients with IPF have Velcro crackles in auscultation, even in patients with limited reduction in FVC, what could suggest that the auscultation of Velcro crackles could be a useful tool to detect early this group of patients.

To our knowledge, this is the first study to assess the potential association between Velcro crackles and different clinical characteristics of ILD. Our study has several strengths. In the first place, it included a large sample size, with prospective collection of all the variables included in the analysis. Second, the results of the multivariate analyses were adjusted for key potential confounders as the ILD-GAP index. Thirdly, all patients were assessed by a multidisciplinary panel of ILD experts. Nevertheless, some limitations of the study need to be considered. In the first place, the assessment of Velcro crackles was performed during the first examination in the ILD unit, so patients with different levels of ILD severity were evaluated. So in the present study we could not assess the usefulness of Velcro crackles as an early sign of ILD or IPF. Interestingly, crackles were auscultated in many patients with initial stages of ILD, especially in IPF where all the patients have Velcro crackles at auscultation. Second, although we could indirectly assume that patients with UIP would have worst prognosis, in our group we did not include variables related to follow-up or long-term prognosis. Third, in the present study we did not assess the extension of Velcro crackles when patients were auscultated. In addition, we did not quantify the extension areas affected in HRCT. In future studies, it might be interesting to explore the correlation of the different areas with Velcro crackles with the extension and type of lesions observed in HRCT. Fourth, although previously to auscultation, an effort was made in order to limit information from the patients, the study could not be completely blinded for physicians. However, the auscultation of Velcro crackles is relatively easy to perform in a clinical setting^5^ and we think that the potential of bias is limited.

In conclusion, in patients with suspicion of ILD, the auscultation of Velcro crackles is associated with a radiological UIP pattern and consequently probably with a more serious prognosis. In addition, all of the patients with IPF have Velcro crackles at auscultation, even in some cases with an FVC >80%. In the absence of validated studies, the auscultation of Velcro crackles in the appropriate clinical setting could be an early warning of the presence of a fibrosing ILD in primary care, although future clinical studies will have to confirm the proper protocol to apply in the early detection of ILDs.
